# Intrinsic Functional Plasticity of the Sensorimotor Network in Relapsing-Remitting Multiple Sclerosis: Evidence from a Centrality Analysis

**DOI:** 10.1371/journal.pone.0130524

**Published:** 2015-06-25

**Authors:** Ying Zhuang, Fuqing Zhou, Honghan. Gong

**Affiliations:** 1 Department of Radiology, the First Affiliated Hospital, Nanchang University, Nanchang, Jiangxi Province, China; 2 Department of Oncology, the Second Hospital of Nanchang, Nanchang, Jiangxi Province, China; 3 Jiangxi Province Medical Imaging Research Institute, Nanchang, Jiangxi Province, China; University of Jaén, SPAIN

## Abstract

**Background and Purpose:**

Advanced MRI studies have revealed regional alterations in the sensorimotor cortex of patients with relapsing-remitting multiple sclerosis (RRMS). However, the organizational features underlying the relapsing phase and the subsequent remitting phase have not been directly shown at the functional network or the connectome level. Therefore, this study aimed to characterize MS-related centrality disturbances of the sensorimotor network (SMN) and to assess network integrity and connectedness.

**Methods:**

Thirty-four patients with clinically definite RRMS and well-matched healthy controls participated in the study. Twenty-three patients in the remitting phase underwent one resting-state functional MRI, and 11 patients in the relapsing-remitting phase underwent two different MRIs. We measured voxel-wise centrality metrics to determine direct (degree centrality, DC) and global (eigenvector centrality, EC) functional relationships across the entire SMN.

**Results:**

In the relapsing phase, DC was significantly decreased in the bilateral primary motor and somatosensory cortex (M1/S1), left dorsal premotor (PMd), and operculum-integrated regions. However, DC was increased in the peripheral SMN areas. The decrease in DC in the bilateral M1/S1 was associated with the expanded disability status scale (EDSS) and total white matter lesion loads (TWMLLs), suggesting that this adaptive response is related to the extent of brain damage in the rapid-onset attack stage. During the remission process, these alterations in centrality were restored in the bilateral M1/S1 and peripheral SMN areas. In the remitting phase, DC was reduced in the premotor, supplementary motor, and operculum-integrated regions, reflecting an adaptive response due to brain atrophy. However, DC was enhanced in the right M1 and left parietal-integrated regions, indicating chronic reorganization. In both the relapsing and remitting phases, the changes in EC and DC were similar.

**Conclusions:**

The alterations in centrality within the SMN indicate rapid plasticity and chronic reorganization with a biased impairment of specific functional areas in RRMS patients.

## Introduction

Multiple sclerosis (MS) is a chronic disease with inflammatory demyelination and neural degeneration. MS is the most common non-traumatic cause of neurological disability in young adults in North America and Europe [1], and the severity of this disease is also a major health concern in China [2]. Most MS patients (approximately 85%) are diagnosed with relapsing-remitting MS (RRMS), which is marked by alternating episodes of disability and recovery. Current pharmacological treatments are aimed at limiting inflammation, decreasing the rate of relapse, and relieving symptoms. During the transition from the relapsing phase to the remitting phase, clinical function may be maintained via central plasticity or reorganization to compensate for (limited) neurological dysfunction [3–6]. Comprehensive knowledge about central plasticity/reorganization in RRMS patients is important for the development of novel therapeutic strategies that may be highly beneficial for these patients. However, because this plasticity or chronic reorganization has not been fully characterized in the relapsing and remitting phases using large-scale imaging in *vivo*; therefore, the exact mechanisms that sustain these phenomena remain unclear.

The analysis of sensorimotor deficits and recovery, which are characteristic of RRMS, provides an opportunity to probe central plasticity or reorganization. These characteristics of MS have previously been described in pathology [7], neuroimaging [8,9], transcranial magnetic stimulation (TMS)[10], and motor training studies [11]. The majority of neuroimaging studies that have assessed sensorimotor plasticity or reorganization in MS were based on structural [8] and functional [11] magnetic resonance imaging (fMRI), which provides the average neural activity during a defined task [12] or in a resting state [13]. In a previous seminal study [14], observations in the remitting phase of RRMS strongly suggested that regional changes (presumably adaptive) had occurred in the sensorimotor cortex (SMC), which were invisible with structural imaging [8,14]. These changes may have been due to an adaptive disability [15,16] or compensatory activation/recruitment [14]. These observations suggest preservation of the functional adaptive reserve in the brain. In the relapsing phase of RRMS, several studies have reported rapid functional plasticity [17–19] accompanied by a loss of neuronal integrity (reduced N-acetylaspartate) [17] in the SMC.

These MRI studies focused on regional disturbances in the sensorimotor network (SMN) [7,20] but ignored the complexity of the functional network or the connectome as a whole. The functional features of network organization and architecture across the entire SMN have not been directly revealed, particularly the features that underlie the relapsing and remitting phases of RRMS. Voxel-wise centrality (including degree [21] and eigenvector centrality [22], DC and EC) is a class of graph theory-based network measurements that assess centrality and functional connectivity, which has received significant attention. DC or EC measurements may be used to determine the functional relationship between a given voxel (node) and the entire connectivity matrix (connectome) rather than between specific nodes or regions [21,22]. In contrast to regional functional (the amplitude of low-frequency fluctuations [23] or regional homogeneity [24]) and hypothesis-driven functional connectivity [25] analyses, this index of network centrality emphasizes network integrity and connectivity. Accordingly, centrality analyses reveal direct (DC) and global (EC) information processing within the SMN without requiring a priori nodes or regions of interest to be selected.

**Fig 1:**
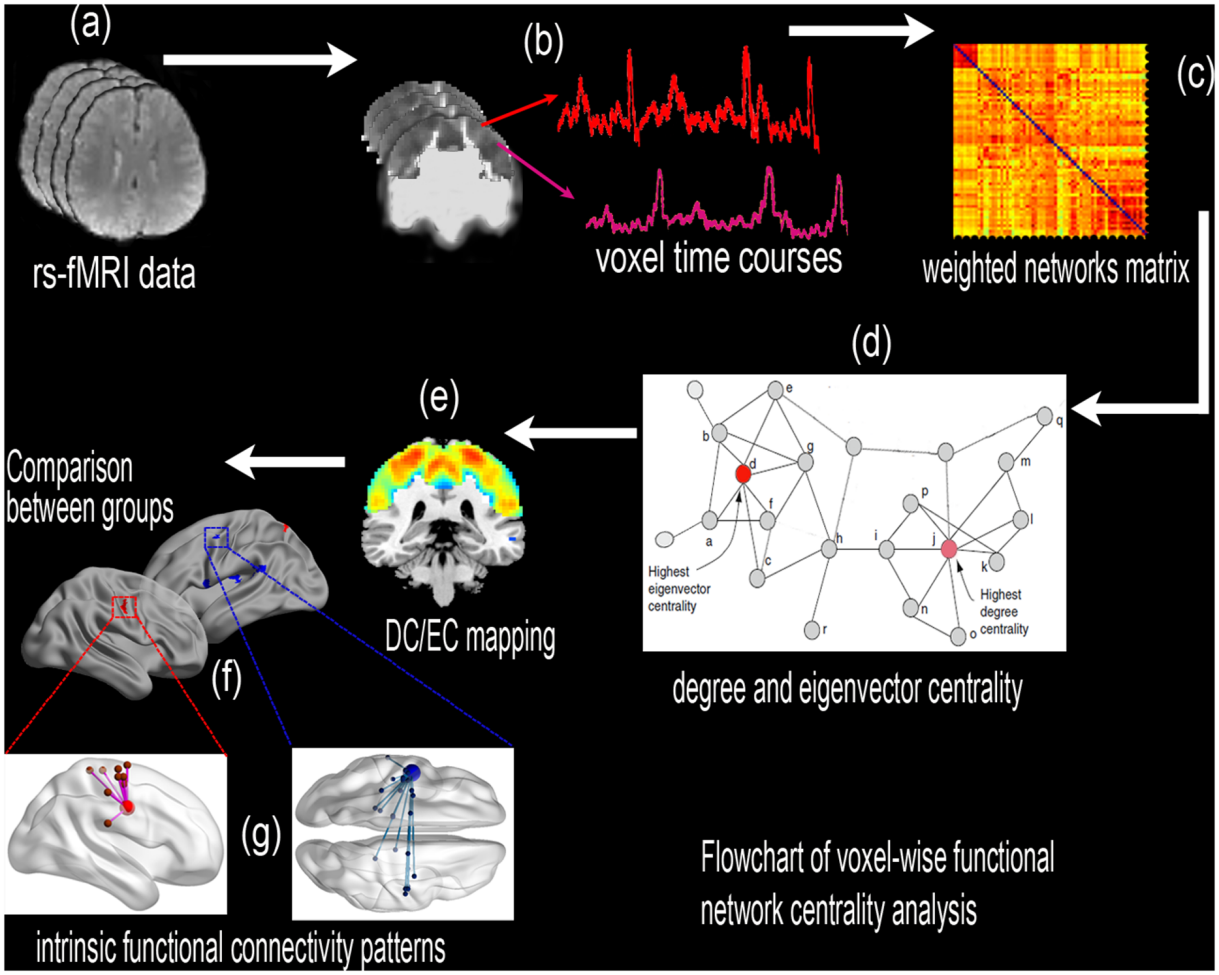
A flowchart of the voxel-wise functional network centrality analysis in the SMN. (a) Preprocessing of the resting-state fMRI data. (b) The time series of each voxel in the SMN template was extracted in MNI space, and (c) *Pearson’s* linear correlation was used to construct the voxel-wise weighted matrix. (d) Voxel-wise degree and eigenvector centrality (DC and EC) were calculated using the “REST-DC” and “fast ECM” toolkits, respectively. (e) The voxel-wise mapping of the SMN. (f) A comparison of network centrality between the groups. (g) Additional intrinsic functional connectivity patterns.

This study used voxel-wise DC and EC mapping to determine the spatial pattern of the sensorimotor network in RRMS patients during the remitting and relapsing phases and to investigate changes in functional connectivity in these patients (Fig 1). First, DC/EC maps were constructed for a group of RRMS patients and healthy controls (HCs). Subsequently, the DC/EC maps were compared between the HCs and the patients in the relapsing and remitting phases. A simple linear regression analysis was conducted for the relapsing and remitting groups to evaluate the correlation between the clinical metrics and the centrality index of abnormal regions. This study could reveal that abnormalities in the connectome of the RRMS patients according to the measure of direct network connectivity (DC) and the sum of centralities of the node’s direct neighbors (EC), which were indexed across the entire SMN. This study also provides novel and deeper insights into the dysfunctional and compensatory mechanisms of RRMS and improves our understanding of the intrinsic functional plasticity or reorganization in these patients.

## Results

### 2.1. Demographic and clinical data

The demographic and clinical characteristics of the RRMS patients and HCs are summarized in Table 1. Significant differences were observed between the patients in the remitting and relapsing phases and the HCs with respect to the brain parenchymal fraction (BPF) values. The BPF is the ratio of brain parenchymal volume to intracranial volume. Significant differences were also found in the expanded disability status scale (EDSS) results between the relapsing and remitting groups, which revealed functional recovery from disability in the remission phase.

**Table 1:** The demographic and clinical characteristics of the study population.

	Relapsing patients *vs*. remitting patients (n = 11)			Remitting patients *vs*. healthy controls (n = 34)		
	Relapsing patients	Remitting patients	*P* values	Remitting patients	Healthy controls	*P* values
**Gender (M/F)**	6/5	6/5	1	13/21	13/21	1
**Mean age (range) (years)**	43.8 (34–57)	43.8 (34–57)	0.95	42.1 (20–58)	41.8 (21–58)	0.96
**Mean disease duration (range) (months)**	20.71 (0.3–72)	28.1 (0.7–72.5)	0.496	26.6 (1.5–150)	-	n/a
**BPF**	0.830±0.004	0.830±0.005	0.968	0.826±0.004	0.861±0.003	0.000
**TWMLL (ml)**	20.36±6.18	19.16±5.84	0.744	18.33±3.19	-	n/a
**Mean EDSS (range)**	3.40 (2–5.5)	2.30 (0–3.5)	0.040	1.97 (0–3.5)	-	n/a
**Mean head motion**	0.046 ± 0.028	0.029 ± 0.012	0.089	0.044 ± 0.020	0.039 ± 0.018	0.266

Note: - = no data; BPF = brain parenchymal fraction; EDSS = expanded disability status scale; F = female; M = male; n/a = not applicable; relapsing patients = multiple sclerosis patients in the relapsing phase; remitting patients = multiple sclerosis patients in the remitting phase; TWMLL = total white matter lesion loads; the same abbreviations are used for all figures and tables.

### 2.2. Spatial distribution of DC/EC maps constructed within the SMN

Using spatial distribution maps, the mean DC values for the SMN in the relapsing patients (n = 11), remitting patients (n = 34) and HCs (n = 34) were identified according to different correlation thresholds (*r*_*0*_ = 0.1, 0.15, 0.2, 0.25, 0.3, 0.35, and 0.4). The results for the DC spatial distribution maps (S1a–S1c Fig) and the between-group differences (S1d and S1e Fig) were highly similar and were not dependent on the different correlation thresholds. We mainly report the results for DC, which were calculated using the weighted sum of the positive correlations (thresholding cutoff = 0.25). The statistical significance of each result was based on a threshold of *P* < 0.001. In this study, fast ECM (S2 Fig) did not require thresholding or binarizing of the connectivity matrix [22].

### 2.3. Alterations in network centrality in the relapsing patients

**Fig 2:**
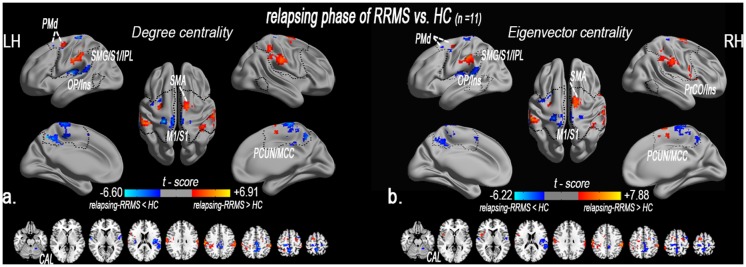
Altered centrality of the sensory-motor network in the relapsing phase of RRMS patients. The spatial distribution of abnormal DC (a) and EC (b) in the relapsing patients compared with the HCs (paired *t*-test, n = 11, *P* < 0.05, AlphaSim corrected critical cluster size k = 20). Spatial distribution was visualized using surface brain imaging in Brainnet Viewer (www.nitrc.org/projects/bnv/). *Note*: *DC = degree centrality; EC = eigenvector centrality; fO = frontal operculum; IPL = inferior parietal lobule; k/k*_*0*_ *= normalized DC; LH = left hemisphere; M1 = primary motor cortex; MNI = Montreal Neurological Institute; MCC = middle cingulate cortex; RRMS = relapsing-remitting multiple sclerosis; PMd = premotor dorsal; RH = right hemisphere; OP/Ins = operculum parietale/insula; PostG = postcentral gyrus; SPL = superior parietal lobule; SMA = supplementary motor area; u/u*_*0*_ *= normalized EC; the same abbreviations are used for all the figures and tables*.

Compared with the healthy controls (n = 11), the RRMS patients who were in the relapsing phase (n = 11) exhibited significantly decreased DC in the bilateral primary motor and somatosensory cortex (M1/S1), the bilateral precuneus/middle cingulate cortex (PCUN/MCC), the left operculum parietale/insula (OP/Ins), the left dorsal premotor (PMd), and the left cerebellum anterior lobe (CAL). In the relapsing patients, DC was significantly increased in the left PMd, the left supramarginal gyrus/S1 (SMG/S1), the right supplementary motor area (SMA), and the right SMG/S1 (S1 Table and Fig 2a).

In addition, EC was decreased in the bilateral M1/S1, the bilateral PCUN/MCC, the left OP/Ins, the left PMd, and the left CAL. However, EC was significantly increased in the left PMd, the left SMG/S1, the right SMA, the right SMG/S1, and the right precentral opercular cortex/insula (PrCO/Ins) (S1 Table and Fig 2b).

### 2.4. The relationship between the clinical indices and centrality in the relapsing patients

In the relapsing patients (n = 11), sample linear regression analyses revealed that decreased DC in the bilateral M1/S1 was negatively correlated with the EDSS (*P* = 0.044) and the TWMLLs (*P* = 0.008). Decreased EC in the left M1/S1 was negatively correlated with the EDSS (*P* = 0.035) and the TWMLLs (*P* = 0.066). In addition, decreased EC in the right M1/S1 was associated with the EDSS (*P* = 0.156) and the TWMLLs (*P* = 0.102). In the relapsing group, the correlation analysis did not reveal significant correlations between the lesion load (TWMLL), brain atrophy (BPF), disease duration, or physical disability (EDSS) and enhanced DC or EC (S2 Table).

### 2.5. A comparison of network centrality between the relapsing and remitting patients

**Fig 3:**
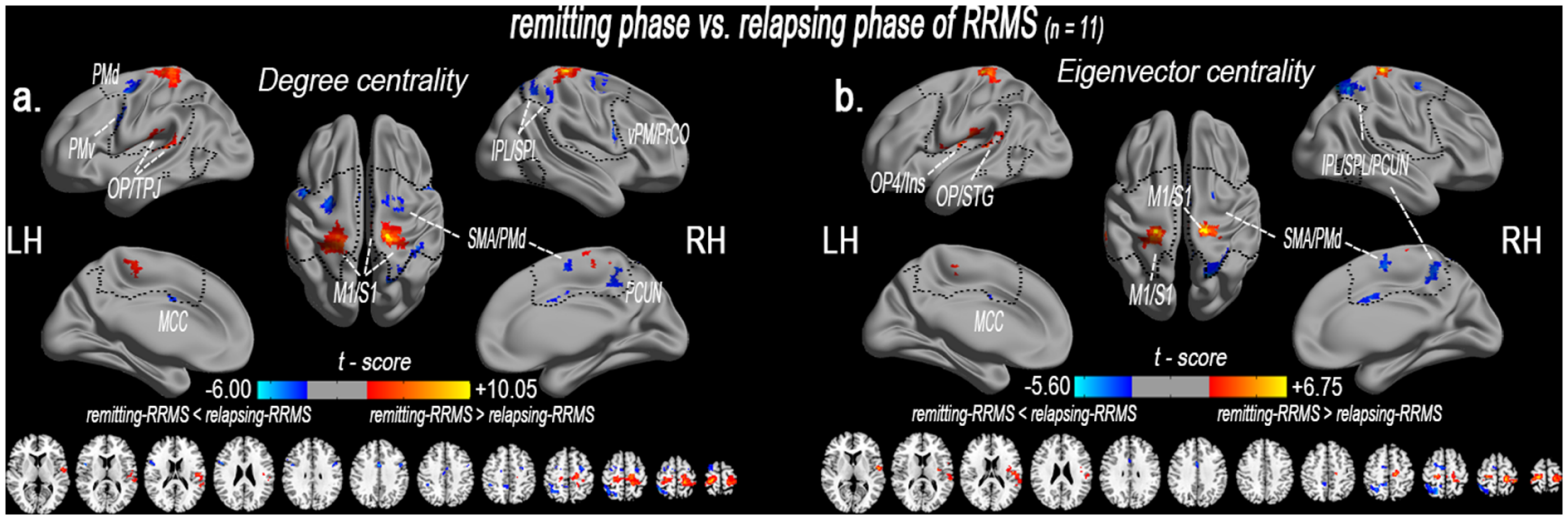
A comparison of network centrality between the remitting and relapsing phase of RRMS patients. Altered degree (a) and eigenvector (b) centrality of the sensory-motor network in the remitting RRMS patients compared with the relapsing RRMS patients (paired *t*-test, n = 11; P < 0.05, AlphaSim corrected critical cluster size k = 20). Network centrality was visualized using surface brain imaging with Brainnet Viewer (www.nitrc.org/projects/bnv/).

Compared with the relapsing patients (n = 11), the remitting patients (n = 11) exhibited significantly decreased DC in the right ventral premotor and PrCO (vPM/PrCO), the left ventral premotor (PMv) and PMd, the bilateral MCC, the right PCUN, the right SMA and the right parietal-integrated regions (inferior and superior parietal lobule, IPL/SPL). DC was significantly decreased in the bilateral M1/S1 and the left rolandic operculum/temporo-parietal junction (OP/TPJ) (S3 Table and Fig 3a).

Additionally, decreased EC was observed in the bilateral MCC, the bilateral supplementary motor area (SMA), and the right IPL/SPL/PCUN. EC was significantly increased in the left operculum-integrated regions (OP/superior temporal gyrus, STG, and OP4/Ins), the left M1/S1 and the right M1/S1 (S3 Table and Fig 3b). S3 Fig displays a comparison of the network centrality within the sensory-motor network between the remitting patients (n = 34) and the relapsing patients (n = 11).

### 2.6. Alterations in network centrality in the remitting patients

**Fig 4:**
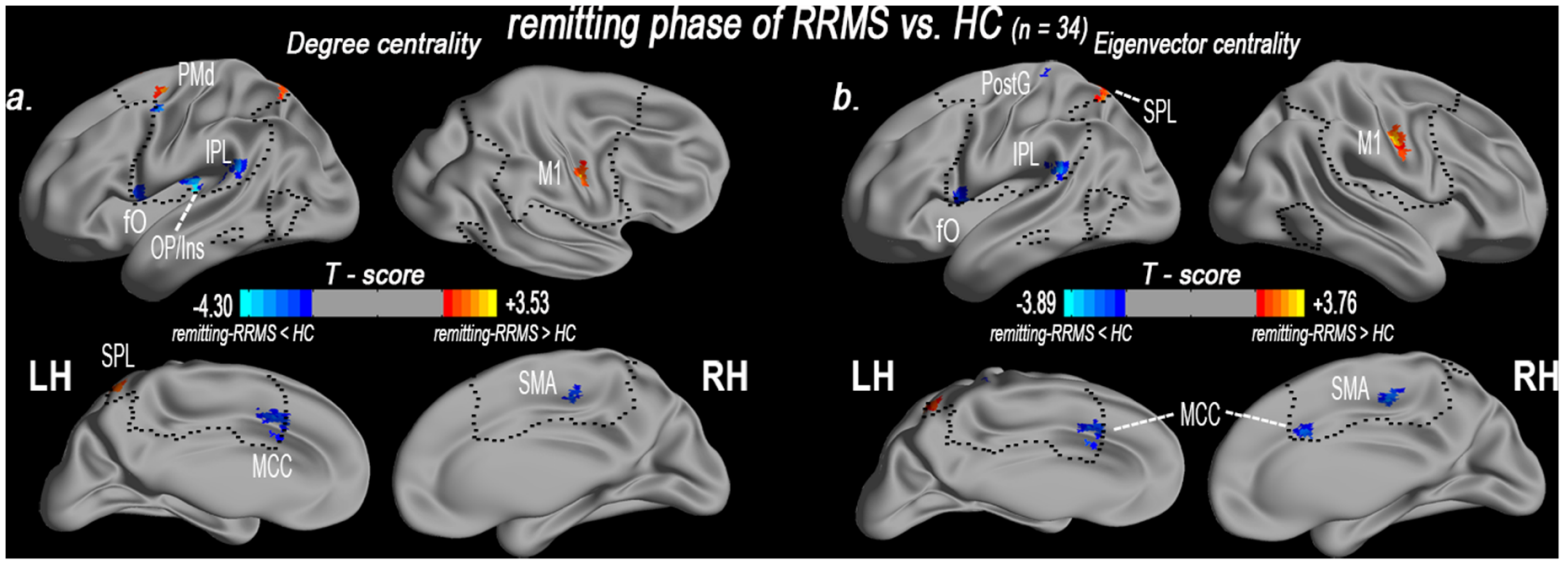
Altered centrality of the sensory-motor network in the remitting phase of RRMS patients. The spatial distribution of abnormal DC (a) and EC (b) in the remitting phase of RRMS patients (*P* < 0.05, AlphaSim corrected critical cluster size k = 20) as visualized using surface brain imaging in Brainnet Viewer (www.nitrc.org/projects/bnv/).

Compared with the HCs (n = 34), the remitting RRMS patients (n = 34) exhibited significantly decreased DC in the left fO, the OP/Ins, the IPL, the left MCC, the left PMd, and the right SMA. DC was significantly increased in the right M1, the left PMd and the left SPL (S4 Table and Fig 4).

Additionally, EC was decreased in the left fO, the left IPL, the bilateral MCC, the right SMA, and the left postcentral gyrus (PostG). However, EC was significantly increased in the right M1 and the left SPL (S4 Table and Fig 4).

### 2.7. The relationship between the clinical indices and centrality in the remitting patients

**Fig 5:**
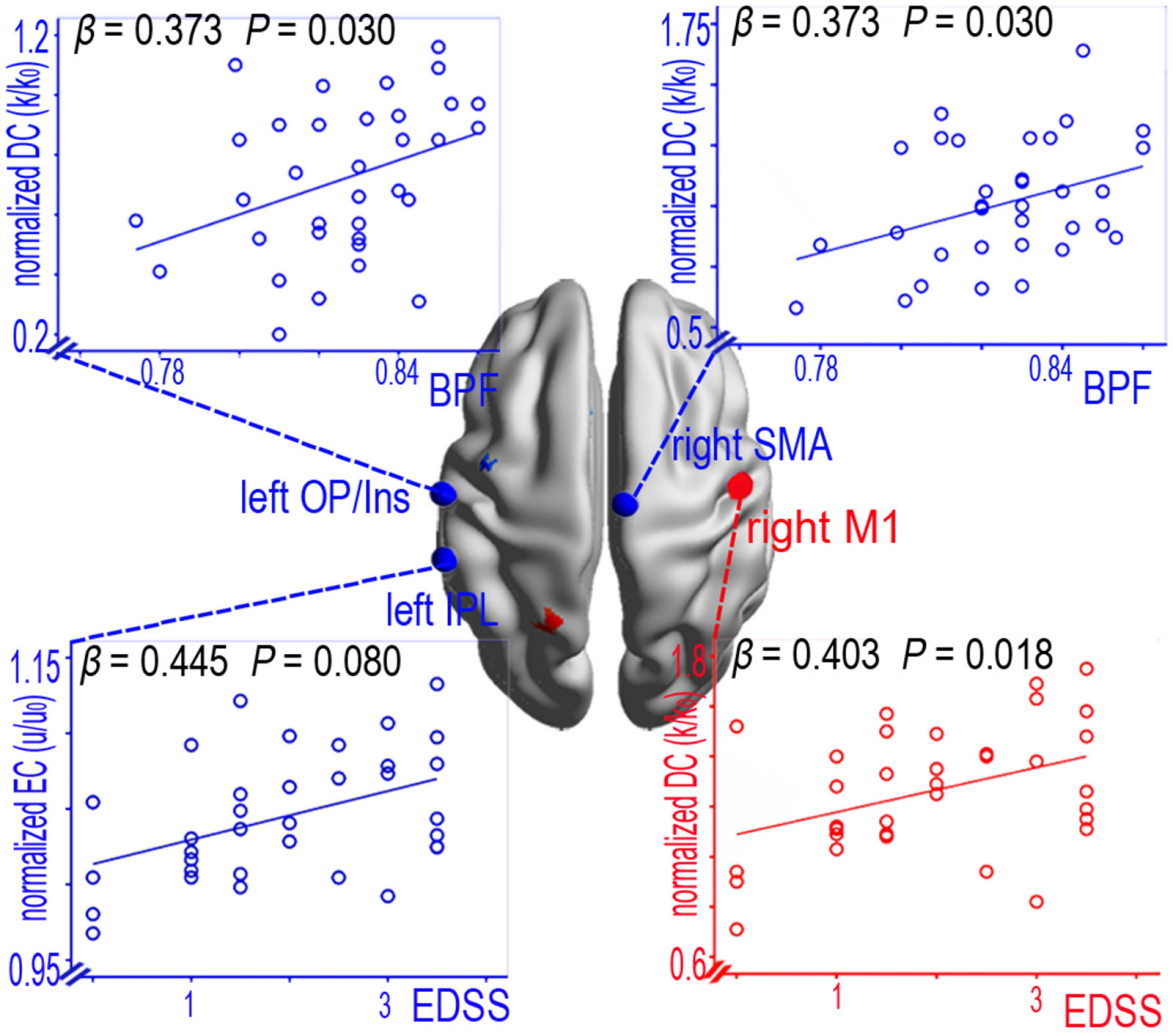
The correlations between the centrality (DC and EC) metrics within the entire SMN and the clinical variables in the remitting phase of RRMS patients.

In the remitting patients (n = 11), linear regression analyses of the lesion load (TWMLL), brain atrophy (BPF), disease duration, physical disability (EDSS) and the DC or EC in the SMN regions revealed significant group differences. Only decreased DC in the left OP/Ins (*P* = 0.030) and the right SMA (*P* = 0.030) were positively correlated with BPF. Increased DC in the right M1 was positively correlated with the EDSS (*P* = 0.018), and decreased EC in the left IPL was positively correlated with the EDSS (*P* = 0.008) (Fig 5). By contrast, no significant relationship was found between the clinical indices and the other abnormal centrality values for the remitting patients (S5 Table).

## Discussion

The primary finding of this study was that the RRMS patients exhibited functionally relevant rapid plasticity and chronic reorganization in the relapsing phase and remitting phase, which may be adaptive mechanisms to maintain sensory and motor function. In the relapsing patients, DC was significantly decreased in the bilateral M1/S1, the bilateral PCUN/MCC, the left OP/Ins, the left PMd, and the left CAL. However, DC was increased in the peripheral SMN areas. In addition, the decrease in DC in the M1/S1 was associated with the EDSS and TWMLLs, which suggest that this adaptive response is related to the extent of brain damage in the rapid-onset attack stage. These alterations in centrality were restored in the bilateral M1/S1 and peripheral SMN areas of the patients who were in the remission process. In the remitting patients, DC was decreased in the premotor, supplementary motor and related integrated regions, which suggests that the adaptive response is related to the extent of brain damage (associated with BPF). However, DC was enhanced in the right M1 and the left SPL, which indicates that the chronic reorganization may have been due to less efficient motor information processing. In the relapsing and remitting phase of RRMS patients, the changes in EC were similar to those of DC.

### 3.1. Evidence of rapid plasticity due to altered centrality in the relapsing phase

The presence of SMN abnormalities (Fig 2 and S1 Table) in the relapsing phase of RRMS patients was not surprising because of previous evidence [26]. This finding suggests functional disconnections in the M1 (or the S1), which provides the descending signals to execute movements or receives dense somatosensory information. In the acute relapsing phase, a negative correlation was observed between decreased DC in the bilateral M1/S1 and the EDSS and TWMLLs. Our data suggest that impaired connectivity in the M1/S1 was due to dysfunction during the acute attack stage.

This study also found functional disconnections in several multimodal integration regions (e.g., premotor, operculum-/cingulate-integrated regions), particularly in OP/Ins. The OP/Ins is the junction region of the insula and operculum areas, and it appears to play a key role in the integrative process between motor and somatosensory functions [27]. The multimodal integration regions were also vulnerable regions in MS according to previous studies[13,28]. Decreased activity levels in multimodal integration regions have been detected in a previous study on the “rubber hand illusion [13,29]” and also exist in cervical myelopathy patients with perception loss [30]. Our data also suggest that impaired connectivity in the multimodal integration regions was an important plastic adaptation due to MS pathology.

Furthermore, this rapid cortical plasticity was involved in enhanced connectivity in the surrounding peripheral regions (e.g., premotor, SMA, and parietal-integrated regions). Functional restoration during the transition from the relapsing phase to the remitting phase may be limited by the impaired expression of rapid-onset synaptic plasticity in RRMS patients [31]. In these patients, this synaptic plasticity, together with remyelination and repair of neuronal damage, are important mechanisms that lead to recovery [32].

These findings, both of loss and of enhanced DC in SMN, indicate that rapid cortical plasticity processes occur during the acute relapsing and remitting phases of RRMS, and that these processes are associated with incomplete symptom recovery.

### 3.2. Evidence of chronic reorganization due to altered centrality in the remitting phase

This study found that the remitting patients exhibited a significant reduction in DC in the premotor (left PMd) and supplementary motor area (right SMA), which are involved in motor control and planning. The left PMd is densely connected to the operculum-integrated pathway [33] (e.g., Ins [34]) and the cingulate motor regions [35], which selectively mediate the concordance of somatosensory-motor information and self-initiated movements. Therefore, these connections may play a key role in selecting appropriate motor plans to achieve desired end results. However, such connections were weaker in the remitting phase of RRMS patients. A similar reduction in functional connectivity (FC) has been observed in the rubber hand illusion, which may be a perceptual illusion caused by multisensory integration in the premotor cortex [29]. Previous structural MRI studies have identified reductions in gray matter and anatomical connectivity [36] in the premotor and supplementary motor area, which suggests morphological impairments in both motor input pathways. Therefore, decreased DC in the left PMd and the right SMA and reduced FC between the operculum-integrated and cingulate motor regions in remitting phase of RRMS patients indicates that neural impairments are the underlying deficits in sensorimotor and sensory-guided actions. These findings provide a basis for understanding decreased excitability and central motor plasticity in the PMd and the SMA during the remitting phase [37].

Compared with the relapsing phase of RRMS patients, DC was more significantly decreased in the sensorimotor-related integrated regions (left fO, left OP/Ins, left IPL, and left MCC) of the remitting patients. These regions share connectivity with multi-regions that are involved in the primary sensory and motor, operculum-/parietal-/cingulate-integrated pathways. The left fO is part of the operculum-integrated pathway, and both the fO and the OP/Ins are crucial for the multimodal receptive fields that are involved in multimodal action recognition [33]. The IPL is involved in the interpretation of sensory information, and the MCC (cingulate motor) is more directly involved in somatic motor control, which plays a crucial role in action initiation [35]. This abnormality has been identified in previous functional and structural MRI studies, which suggests that the sensorimotor-related integrated pathway plays a neuro-modulatory role in biasing sensor-motor-spatial processing and modulation [6,15,38]. In this study, the decrease in DC in the left OP/Ins and the right SMA was positively correlated with the BPF. In contrast to the lesion load, brain atrophy measurements can provide an estimate of the amount of tissue destruction due to chronic pathological processes [39,40]. These results suggest that cortical adaptive responses may play an important role in the chronic reorganization process in RRMS.

In addition, we found that DC was significantly increased in the primary motor (right M1), the premotor (left PMd) and the parietal integrated pathway (left SPL). The increase in DC in the right M1 was positively correlated with the EDSS. Bodini et al. [41] have suggested that T2 brain lesions are an important contributor to the progression of disability, and a recent resting-state functional MRI (rs-fMRI) study revealed that increased functional connectivity was associated with the severity of impairment [42]. Recent evidence suggests that synapses in the M1 may result in dynamic plasticity following pathological changes to facilitate cognitive and motor activities [43]. This study’s finding of increased functional connectivity in the remitting phase of RRMS patients indicates less efficient neuronal processing in the executive frontal-motor-parietal integrated pathway.

### 3.3. Similarity alteration between degree and eigenvector centrality of sensorimotor network

In this study, the distribution changes in EC were similar to the changes in DC in both the relapsing and remitting phases of RRMS patients, when compared with the HCs. DC is primarily a local measurement of the connectome graph, which is used to index the number of direct connections and direct functional relationships for a given node [21]. EC is a relative global measurement that indexes the qualitative superiority of node connections rather than the number of direct connections, which provides global information processing [21,22]. The population of voxels exhibited relatively high correlation between EC and DC. Relative to DC, EC demonstrated significantly higher centrality for subcortical regions but similar distribution patterns within SMN. The decreased functional connectivity[13] and EC [44] in sensorimotor activity associated with physical disability was demonstrated in MS patients. We interpret these findings regarding the similarity alteration between DC and EC within the sensorimotor network as supporting the hypothesis that although the direct connectivity of these regions decreases with MS-related damage, it also affects their connections at a global level.

This study had several technical and biological limitations. First, the acquired images were limited in spatial resolution due to a 4-mm slice thickness. Future studies that use higher-resolution fMRI should be implemented. Second, the number of paired RRMS patients was relatively low; therefore, a larger sample should be used in future studies. Third, this study lacked a related assessment of sensorimotor functional integration in the participants. Finally, this study focused on alterations in the SMN, and the SMN mask was obtained from HCs using the Group ICA Toolbox (http://mialab.mrn.org/data/index.html), which includes the classical sensorimotor cortex and related integrated regions. Other SMN masks may identify different DC and SC levels in undiscovered regions. However, using a different mask may not affect the overall results reported in this study. The comparison of functional centrality among the groups was independent of the image mask, excluding the voxels near the mask boundary.

## Conclusion

This study characterized the intrinsic functional plasticity or reorganization in RRMS patients using DC and EC mapping. The findings expand our understanding of the functional characteristics of RRMS, which include relatively reduced centrality (e.g., left OP/Ins) in response to the destructive aspects of MS and relatively enhanced centrality (e.g., right M1) that acts as a functional adaptive reserve. An intriguing speculation is that these functional integrated regions may be more decompensated in the chronic remitting phase, which may have implications for the treatment of sensorimotor function in RRMS patients.

## Experimental Procedures

### 5.1. Subjects

We recruited 34 patients with clinically definite RRMS according to McDonald’s criteria [1] at the First Affiliated Hospital of Nanchang University from May 2010 to December 2013. The inclusion criteria for the patients included an RRMS course [45] and a history of treatment with immunomodulatory medication (20 with β-interferons and 4 with glatiramer acetate). In this study, 23 patients underwent one MRI scan during the remitting phase, and 11 patients underwent two MRI scans-one scan each during the relapsing and remitting phases, respectively. Because RRMS patients who have relapses usually recover within 2–3 months after onset, we scheduled a follow-up neurological assessment and MRI 12 weeks after relapse onset. Thirty-four HC participants from the local community were individually matched with the patients for sex, age, and education level. The control patients had no history of hypertensive disease, traumatic brain injuries, neurology diseases, or brain abnormalities based on conventional MRI scans. This study was approved by the Medical Research Ethics Committee and the Institutional Review Board of the First Affiliated Hospital of Nanchang University. This study was performed according to approved guidelines and was conducted in compliance with the principles of the Declaration of Helsinki. All of the patients signed written consent forms before participating in the study. The detailed demographic and clinical data are displayed in Table 1.

### 5.2. Image acquisition

All participants underwent MRI scans using a 3.0 T MRI scanner (Trio Tim, Siemens Medical Systems, Erlangen, Germany). The rs-fMRI images were acquired using the echo planar imaging (EPI) sequence (repetition time [TR]/echo time [TE] = 2,000/30 ms; flip angle = 90°; field of view [FOV] = 200 × 200 mm; matrix = 64 × 64; 30 interleaved axial slices, 4-mm thickness with an interslice gap of 1.2 mm; and 240 functional volumes for each patient). In addition, the scan consisted of three-dimensional high-resolution *T*_1_-weighted imaging (T_1_WI) (TR/TE = 1,900/2.26 ms; matrix = 240 × 256; FOV = 215 × 230 mm; number of excitations [NEX] = 1; 176 sagittal slices with a 1.0-mm slice thickness) and *T*_2_-weighted turbo spin echo imaging (TR/TE = 5,100/117 ms; NEX = 3; echo train length = 11; matrix = 416 × 416; FOV = 240 × 240 mm; slice number = 22; slice thickness = 6.5 mm; and orientation = axial). During rs-fMRI scanning, the patients were instructed to keep their eyes closed, to avoid thinking systematically, and not to fall asleep. A foam pad was used to minimize the head motion of all of the patients.

### 5.3. Preprocessing of fMRI data

The fMRI data were preprocessed using the Data Processing Assistant for Resting-State fMRI Advanced Edition (DPARSFA) V2.3 (http://www.restfmri.net), which was run on the Matlab 2012a (MathWorks, Inc., Natick, MA, USA) platform. Briefly, the first 10 functional volumes were discarded to allow for stabilization of the initial signal and to eliminate magnetic saturation effects. The remaining fMRI images were processed with slice time correction for interleaved slice acquisition and three-dimensional motion corrections. No patient was excluded based on head motion criteria, which included cardinal directions (x, y, z) of less than 2 mm and a maximum spin (x, y, z) of less than 2°. We also respectively evaluated the group differences in head motion among the relapsing patients (n = 11), remitting patients (n = 34) and HCs (n = 34) according to the criteria of Van Dijk et al. [46]. The results indicated that the 3 groups displayed no significant differences in head motion (1-way analysis of variances [ANOVAs] with Bonferroni-corrected post hoc t tests, *P* = 0.451; Table 1). Then, the high-resolution individual T_1_WI images were coregistered to the mean functional image after motion correction using a linear transformation, and the images were segmented into gray matter (GM), white matter, and cerebrospinal fluid tissue maps using a priori SPM tissue maps as a reference and a unified segmentation algorithm [47]. The resultant GM, white matter, and cerebrospinal fluid images were further nonlinearly registered in Montreal Neurological Institute (MNI) space using the estimates in the unified segmentation, and the images were averaged across all the patients to create custom GM, white matter, and cerebrospinal fluid templates (also for the measurement of brain atrophy). Next, the coregistered T1 images were segmented again using the custom tissue templates as reference images and the unified segmentation algorithm [47] to reduce the risk of inaccuracy in the spatial normalization of the functional volumes due to GM atrophy. Then, we resampled the transformational functional images to 3-mm cubic voxels. Spatial smoothing using a full-width half-maximum Gaussian kernel (FWHM-6 mm) and temporal band-pass filtering (0.01–0.08 Hz) was used to reduce low-frequency drift and physiological high-frequency noise. Finally, nuisance linear regression was performed using white matter, cerebrospinal fluid, and six head motion parameters as covariates. The residuals were used for the network centrality analysis.

### 5.4. Voxel-wise degree and eigenvector centrality analysis

DC values were obtained in MNI space for the resting-state fMRI time series using the “REST-DC” toolkit in the REST V1.8 package (http://www.restfmri.net)[21]. A voxel-wise measurement of ‘‘degree centrality” was captured for the weighted networks in terms of graph theory. First, a voxel-wise (*Pearson’s* linear) correlation matrix was computed within the sensory-motor network mask (N voxels = 14,106), and a functional template was generated in an independent component analysis (ICA) from the “Medical Image Analysis (MIA) Lab (http://mialab.mrn.org/data/index.html)”. We then calculated the voxel-wise DC using the following equation:

$$DC=\sum_{j=1}^{N}k_{voxel}(i)=\sum_{j=1}^{N}r_{ij}{}_{\left(r_{ij}>r_{0}\right)}$$ (1)

where r_ij_ is the correlation coefficient between voxel i and voxel j and r_0_ is a threshold that is set to eliminate weak correlations [21,48,49]. Different *r*_*0*_ values (*r*_*0*_ = 0.1, 0.15, 0.2, 0.25, 0.3, 0.35, and 0.4) were considered in this study. The *k (i)* of each voxel was divided by the individual global mean of *k*_*0*_ within the SMN mask to normalize the values and to reduce the effect of individual variability. Then, the individual data were converted using Fisher’s Z-transformation for the group comparisons [21,50]. DC is a characterization of the strength of functional connectivity of a voxel with respect to the observational whole network, which is used to index the number of direct connections for a given node. DC is the most directly quantifiable centrality measurement.

Voxel-wise EC values within the sensorimotor network were obtained using a fast ECM toolkit (https://code.google.com/p/bias/source/browse/matlab/fastECM) [22]. Similarly to degree centrality, EC relies on the assumption that each node's centrality is the sum of the centrality values of the nodes to which it is connected. However, in contrast to degree centrality, EC specifically favors nodes that are connected to other central nodes within the network. EC was defined as follows:

$$EC\left(i\right)=\mu_{1}\left(i\right)=\frac{1}{\lambda_{1}}A\mu_{1}=\frac{1}{\lambda_{1}}\sum_{j=1}^{N}a_{ij}\mu_{1}(j)$$ (2)

where λ_1_ is the largest (principal) eigenvalue. Because fast ECM does not require thresholding or binarizing of the connectivity matrix, all of the patient networks had the same topology and were the same size [22]. The EC value of each voxel was normalized using normally distributed form centrality (tied) ranks. The *u(i)* of each voxel was divided by the individual global mean of *u*_*0*_ within the SMN mask to normalize the values and to reduce the effect of individual variability. Then, the individual data were converted using Fisher’s Z-transformation for the group comparisons.

A comparison of the DC and EC maps was made using a general linear model (GLM), standard statistical parametric mapping (SPM8, http://www.fil.ion.ucl.ac.uk/spm), and one-way analysis of covariance (ANCOVA). Age and gender were used as covariates. Post hoc, two-sample *t*-tests were performed using the SMN mask. The significance level was set at a corrected *P* value < 0.05. Multiple comparisons were corrected using Monte Carlo simulations and the AlphaSim program with the REST package and the following parameters: for individual voxels, *P* = 0.01, FWHM = 6 mm, rmm = 5, and iterations = 1,000.

Sample linear regression (in SPSS) was performed to assess the relationship between the clinical metrics (EDSS, BPF and TWMLL) and the voxel-wise centrality of the obtained regions with significant group differences, controlling for gender and age. All statistical analyses were performed using SPSS with a statistical significance level of *P* < 0.05, and the analyses were corrected for multiple comparisons using the Bonferroni correction.

### 5.5. Measurements of brain atrophy and white matter lesions

The brain parenchymal fraction (BPF), which is the ratio of brain parenchymal volume to intracranial volume, was used to calculate brain atrophy.

An experienced neuroradiologist manually marked hyperintense white matter lesions on *T*_*2*_-weighted images, which is a common practice in brain imaging studies. Subsequently, each individual binary lesion mask was coregistered and normalized to MNI space for standardization. The inter-rater reliability of the TWMLLs was 93.8% (on two separate occasions at least three months apart).

## Supporting Information

S1 Dataset Altered degree centrality of SMN in the relapsing patients (including demographic and clinical data). (ZIP)

S2 Dataset Altered degree centrality of SMN between the relapsing and remitting patients. (ZIP)

S3 Dataset Altered eigenvector centrality of SMN in the relapsing patients. (ZIP)

S4 Dataset Altered eigenvector centrality of SMN between the relapsing and remitting patients. (ZIP)

S1 Fig Within- and between-group DC maps derived at different correlation thresholds (r_0_). (a-c) Mean DC maps within the RRMS and HC groups using different correlation thresholds (*r*_*0*_ = 0.1, 0.15, 0.2, 0.25, 0.3, 0.35, and 0.4). (d-e) Significant differences were observed in the maps between the 2 groups. Notably, a similar pattern was observed in most of the regions that showed MS-related changes in DC derived at different thresholds.
(TIF)

S2 Fig Group-wise spatial distribution expressed as the mean z-values for eigenvector centrality (EC) within the sensory-motor network of the relapsing and remitting RRMS patients and the healthy controls. (TIF)

S3 Fig Altered centrality of sensory-motor network between the remitting phase (n = 34) and relapsing phase (n = 11) of RRMS patients. Altered degree (a) and eigenvector (b) centrality in the sensory-motor network of the remitting phase compared with the relapsing phase of RRMS patients (two-sample *t*-tests; *P* < 0.05, AlphaSim corrected critical cluster size k = 20) as visualized using surface brain imaging in Brainnet Viewer (www.nitrc.org/projects/bnv/).
(TIF)

S1 Table Significant differences in SMN DC/EC between the relapsing phase of RRMS patients and the HCs. (DOC)

S2 Table Altered centrality associated with the clinical metrics in the relapsing patients. (DOC)

S3 Table Significant differences in SMN DC/EC between the relapsing and remitting phase of RRMS patients. (DOC)

S4 Table Significant differences in SMN DC/EC between the remitting phase of RRMS patients and the HCs. (DOC)

S5 Table Altered centrality associated with the clinical metrics in the remitting patients. (DOC)
