# Supplementary figures and images for: Intrinsic Functional Plasticity of the Sensorimotor Network in Relapsing-Remitting Multiple Sclerosis: Evidence from a Centrality Analysis
Source: PLoS One. 2015 Jun 25;10(6):e0130524. doi: 10.1371/journal.pone.0130524 (PMC4482320; doi:10.1371/journal.pone.0130524)

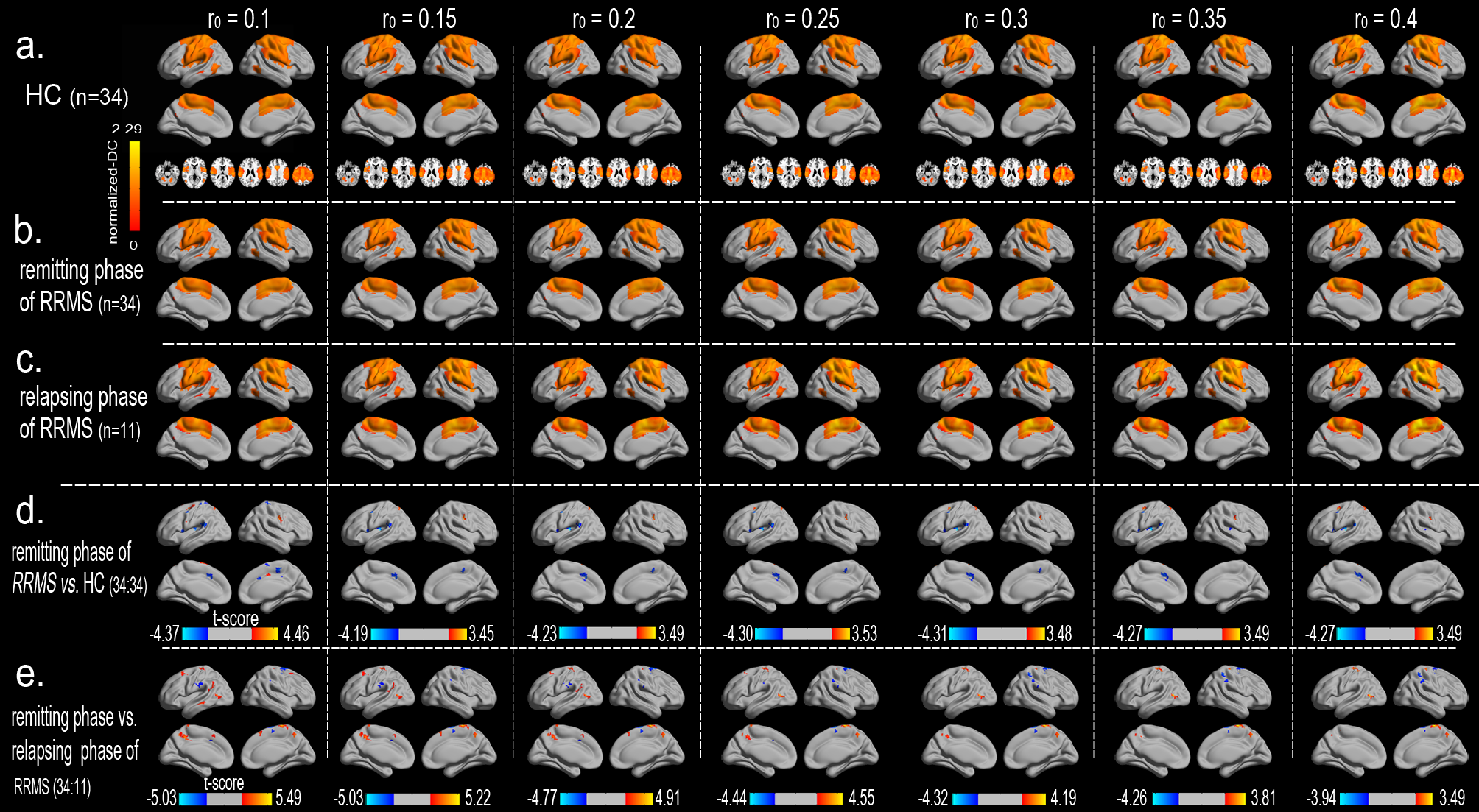

Supplement: S1 Fig — (a-c) Mean DC maps within the RRMS and HC groups using different correlation thresholds (r 0 = 0.1, 0.15, 0.2, 0.25, 0.3, 0.35, and 0.4). (d-e) Significant differences were observed in the maps between the 2 groups. Notably, a similar pattern was observed in most of the regions that showed MS-related changes in DC derived at different thresholds. (TIF) [file pone.0130524.s005.tif]

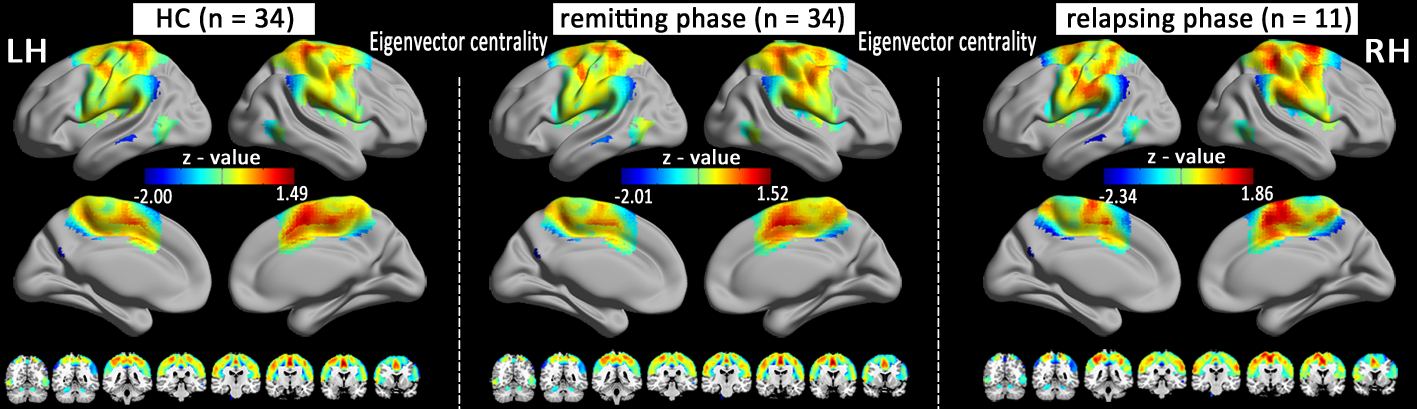

Supplement: S2 Fig — (TIF) [file pone.0130524.s006.tif]

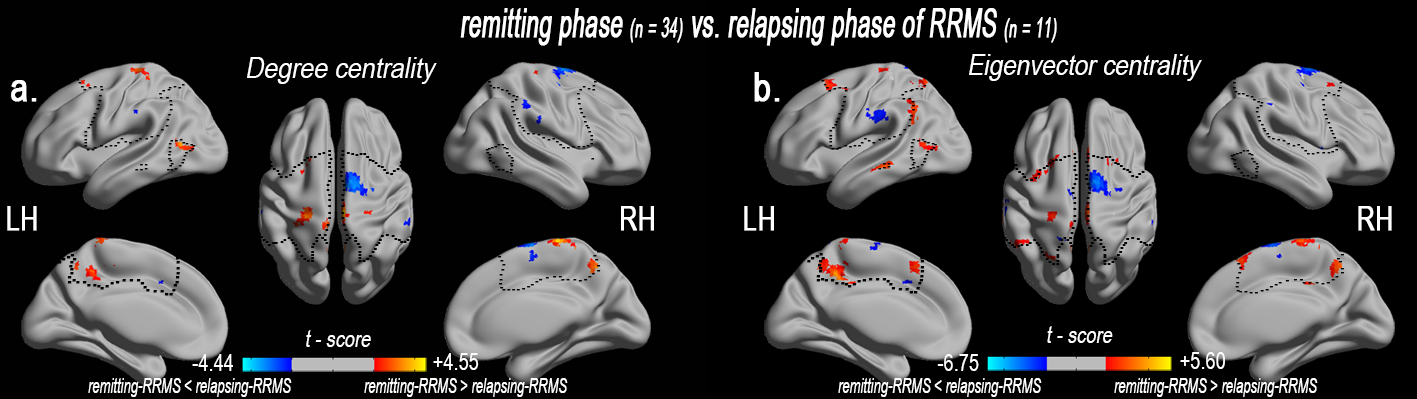

Supplement: S3 Fig — Altered degree (a) and eigenvector (b) centrality in the sensory-motor network of the remitting phase compared with the relapsing phase of RRMS patients (two-sample t-tests; P < 0.05, AlphaSim corrected critical cluster size k = 20) as visualized using surface brain imaging in Brainnet Viewer (www.nitrc.org/projects/bnv/). (TIF) [file pone.0130524.s007.tif]
