# Supplementary material for: Intrinsic Functional Plasticity of the Sensorimotor Network in Relapsing-Remitting Multiple Sclerosis: Evidence from a Centrality Analysis
Source: PLoS One. 2015 Jun 25;10(6):e0130524. doi: 10.1371/journal.pone.0130524 (PMC4482320; doi:10.1371/journal.pone.0130524)
Supplement: S1 Table — (DOC) [file pone.0130524.s008.doc]

**S1 Table.** **Significant differences** **in SMN DC/EC between the relapsing phase of RRMS patients and the HCs.**

| Brain regions |  | BA | Peak T-scores | MNI coordinates | | | Cluster size (voxels) |
| --- | --- | --- | --- | --- | --- | --- | --- |
|  |  |  |  | x | y | z |  |
| Degree centrality: relapsing patients *vs.* HCs | | | | | | | |
| CAL | L |  | -3.67 | -24 | -48 | -30 | 21 |
| OP/Ins | L | 13, 40 | -6.60 | -30 | -24 | 12 | 313 |
| PCUN/MCC | B | 5, 6, 7 | -6.59 | -3 | -57 | 51 | 622 |
| PMd | L | 6 | -3.25 | -39 | -3 | 51 | 34 |
| M1/S1 | L | 4, 3 | -6.13 | -21 | -30 | 69 | 72 |
| SMG/S1 | L | 40, 2 | 6.91 | -60 | -30 | 48 | 231 |
| SMG/S1 | R | 40, 3 | 6.56 | 66 | -27 | 42 | 334 |
| PMd | L | 6 | 4.04 | -18 | 0 | 48 | 56 |
| SMA | R | 6 | 3.56 | 18 | -6 | 69 | 59 |
| Eigenvector centrality: relapsing patients *vs.* HCs | | | | | | | |
| CAL | L |  | -3.75 | -24 | -48 | -30 | 21 |
| OP/Ins | L | 13, 40 | -5.93 | -57 | -33 | 15 | 304 |
| PCUN/MCC | B | 5, 6, 7 | -6.22 | 0 | -51 | 63 | 284 |
| SMA | L | 6 | -3.74 | 0 | 6 | 63 | 23 |
| PMd | L | 6 | -4.34 | -39 | -3 | 51 | 46 |
| M1/S1 | B | 3, 4, 6 | -6.13 | -21 | -30 | 69 | 281 |
| PrCO/Ins | R | 44, 13 | 4.67 | 45 | 3 | 0 | 94 |
| SMG/S1 | L | 40, 2 | 7.88 | -63 | -33 | 45 | 207 |
| SMG/S1 | R | 40, 4, 2 | 6.05 | 66 | -27 | 42 | 328 |
| SMA | R | 6 | 5.29 | 9 | -3 | 66 | 132 |
| PMd | L | 6 | 5.26 | -18 | 0 | 54 | 50 |

*Note: CAL = cerebellum anterior lobe; IPL = inferior parietal lobule; PCUN = precuneus; SMG =* *supramarginal gyrus; STG =* *superior temporal gyrus; the same abbreviations are used for all of the figures and tables.*
