# Supplementary material for: Intrinsic Functional Plasticity of the Sensorimotor Network in Relapsing-Remitting Multiple Sclerosis: Evidence from a Centrality Analysis
Source: PLoS One. 2015 Jun 25;10(6):e0130524. doi: 10.1371/journal.pone.0130524 (PMC4482320; doi:10.1371/journal.pone.0130524)
Supplement: S2 Table — (DOC) [file pone.0130524.s009.doc]

**S2 Table. Altered centrality associated with the clinical metrics in the relapsing patients.**

| Regions | Disease duration | | |  | | EDSS | | | TWMLL | | | BPF | | |
| --- | --- | --- | --- | --- | --- | --- | --- | --- | --- | --- | --- | --- | --- | --- |
|  | *R^2^* | *P* | *β* | | *R^2^* | | *P* | *β* | *R^2^* | *P* | *β* | *R^2^* | *P* | *β* |
| Altered degree centrality in the relapsing patients | | | | | | | | | | | | | | |
| Left OP/TPJ | 0.003 | 0.864 | 0.059 | | 0.011 | | *0.755* | *-0.017* | *0.062* | 0.462 | 0.248 | 0.122 | 0.292 | 0.349 |
| Right vPM/PrCO | 0.205 | 0.162 | -0.453 | | 0.003 | | 0.869 | -0.057 | 0.012 | 0.750 | 0.109 | 0.001 | 0.926 | -0.032 |
| Left PMv | **0.490** | **0.016** | **-0.700** | | 0.041 | | 0.551 | -0.202 | 0.017 | 0.702 | -0.131 | 0.092 | 0.366 | -0.303 |
| Bilateral MCC | 0.304 | 0.079 | 0.551 | | 0.015 | | 0.720 | 0.123 | 0.098 | 0.348 | 0.313 | 0.013 | 0.736 | -0.115 |
| Right PCUN | 0.227 | 0.138 | 0.476 | | 0.100 | | 0.344 | 0.316 | 0.273 | 0.099 | 0.522 | 0.120 | 0.269 | 0.347 |
| Left PMd | 0.241 | 0.125 | -0.491 | | 0.083 | | 0.390 | 0.288 | 0.129 | 0.277 | 0.360 | 0.202 | 0.162 | 0.450 |
| Bilateral M1/S1 | 0.003 | 0.877 | -0.053 | | **0.378** | | **0.044** | **-0.615** | **0.555** | **0.008** | **-0.745** | 0.039 | 0.562 | -0.197 |
| Right IPL/SPL | 0.023 | 0.658 | -0.151 | | 0.040 | | 0.556 | -0.200 | 0.032 | 0.596 | 0.180 | 0.325 | 0.067 | 0.570 |
| Right SMA/PMd | 0.102 | 0.339 | 0.319 | | 0.064 | | 0.453 | 0.253 | *0.086* | 0.381 | 0.294 | 0.345 | 03058 | 0.587 |
| Altered eigenvector centrality in the relapsing patients | | | | | | | | | | | | | | |
| Left OP/STG | 0.271 | 0.101 | 0.520 | | 0.006 | | *0.824* | *0.076* | *0.022* | 0.664 | 0.148 | 0.002 | 0.896 | 0.045 |
| Left OP4/Ins | 0.006 | 0.822 | 0.077 | | 0.012 | | *0.744* | *0.111* | *0.054* | 0.493 | 0.232 | 0.017 | 0.705 | 0.129 |
| Bilateral MCC | 0.101 | 0.341 | 0.318 | | 0.011 | | 0.755 | -0.107 | 0.034 | 0.586 | 0.185 | 0.019 | 0.689 | 0.136 |
| Left M1/S1 | 0.031 | 0.607 | 0.175 | | **0.405** | | **0.035** | **-0.636** | 0.327 | 0.066 | -0.572 | 0.022 | 0.666 | 0.147 |
| Right IPL/SPL/PCUN | 0.0001 | 0.975 | -0.011 | | 0.050 | | 0.507 | -0.225 | 0.008 | 0.796 | 0.089 | 0.075 | 0.416 | 0.274 |
| Bilateral SMA | 0.131 | 0.274 | 0.362 | | 0.169 | | 0.209 | -0.411 | 0.073 | 0.421 | -0.271 | 0.117 | 0.303 | 0.342 |
| Right M1/S1 | 0.015 | 0.720 | 0.122 | | 0.211 | | 0.156 | -0.459 | 0.269 | 0.102 | -0.519 | 0.015 | 0.724 | 0.121 |
