# Supplementary material for: Intrinsic Functional Plasticity of the Sensorimotor Network in Relapsing-Remitting Multiple Sclerosis: Evidence from a Centrality Analysis
Source: PLoS One. 2015 Jun 25;10(6):e0130524. doi: 10.1371/journal.pone.0130524 (PMC4482320; doi:10.1371/journal.pone.0130524)
Supplement: S3 Table — (DOC) [file pone.0130524.s010.doc]

**S3 Table. Significant differences in SMN DC/EC between the relapsing and remitting phase of RRMS patients.**

| Brain regions |  | BA | Peak T-scores | MNI coordinates | | | Cluster size (voxels) |
| --- | --- | --- | --- | --- | --- | --- | --- |
|  |  |  |  | x | y | z |  |
| Degree centrality: relapsing patients *vs.* remitting patients | | | | | | | |
| OP/TPJ | L | 42, 43, 40 | -4.94 | -48 | -18 | 18 | 132 |
| vPM/PrCO | R | 44 | 4.35 | 45 | 6 | 27 | 47 |
| PMv | L | 6 | 4.19 | -48 | 6 | 33 | 37 |
| MCC | B | 24 | 5.40 | 0 | 3 | 36 | 42 |
| PCUN | R |  | 3.30 | 9 | -45 | 57 | 32 |
| PMd | L | 6 | 4.02 | -27 | -3 | 51 | 55 |
| M1/S1 | B | 2, 3, 4 | -10.05 | 18 | -30 | 72 | 628 |
| IPL/SPL | R | 7, 40 | 5.40 | 24 | -60 | 69 | 153 |
| SMA/PMd | R | 6 | 3.38 | 24 | -6 | 72 | 80 |
| Eigenvector centrality: relapsing patients *vs.* remitting patients | | | | | | | |
| OP/STG | L | 22 | -5.74 | -57 | -9 | 6 | 28 |
| OP4/Ins | L | 40, 42 | -4.33 | -36 | -12 | 15 | 143 |
| MCC | B | 24 | 3.75 | 3 | 3 | 36 | 36 |
| M1/S1 | L | 2, 3, 4 | -6.75 | -21 | -36 | 63 | 238 |
| IPL/SPL/PCUN | R | 7, 5 | 5.60 | 21 | -66 | 57 | 211 |
| SMA | B | 6 | 4.76 | 9 | -6 | -6 | 57 |
| M1/S1 | R | 3, 4 | -6.72 | 15 | -30 | 72 | 123 |
