# Supplementary material for: Intrinsic Functional Plasticity of the Sensorimotor Network in Relapsing-Remitting Multiple Sclerosis: Evidence from a Centrality Analysis
Source: PLoS One. 2015 Jun 25;10(6):e0130524. doi: 10.1371/journal.pone.0130524 (PMC4482320; doi:10.1371/journal.pone.0130524)
Supplement: S4 Table — (DOC) [file pone.0130524.s011.doc]

**S4 Table. Significant differences in SMN DC/EC between the remitting phase of RRMS patients and the HCs.**

| Brain regions |  | BA | Peak T-scores | MNI coordinates | | | Cluster size (voxels) |
| --- | --- | --- | --- | --- | --- | --- | --- |
|  |  |  |  | x | y | z |  |
| Degree centrality: remitting patients *vs.* HCs | | | | | | | |
| fO | L | 44 | -3.58 | -57 | 3 | 3 | 43 |
| OP/Ins | L | 13 | -4.30 | -39 | -18 | 12 | 36 |
| IPL | L |  | -3.47 | -63 | -42 | 21 | 46 |
| MCC | L | 32 | -3.34 | -12 | 9 | 45 | 54 |
| SMA | R | 6 | -3.37 | 12 | -24 | 51 | 27 |
| PMd | L | 6 | -3.72 | -45 | -3 | 54 | 28 |
| M1 | R | 4 | 3.31 | 48 | -9 | 36 | 28 |
| PMd | L | 6 | 2.58 | -21 | -6 | 60 | 20 |
| SPL | L | 7, 40 | 3.33 | -33 | -66 | 57 | 60 |
| Eigenvector centrality: remitting patients *vs.* HCs | | | | | | | |
| fO | L | 44 | -3.76 | -57 | 3 | 3 | 40 |
| IPL | L |  | -3.88 | -66 | -42 | 21 | 56 |
| MCC | B | 32 | -3.02 | -15 | 9 | 45 | 73 |
| SMA | R | 6 | -3.24 | 9 | -24 | 54 | 31 |
| PostG | L | 3 | -3.06 | -27 | -45 | 75 | 27 |
| SPL | L | 7 | 3.08 | -24 | -63 | 57 | 44 |
| M1 | R | 4 | 3.76 | 48 | -9 | 36 | 66 |
