# Supplementary material for: Intrinsic Functional Plasticity of the Sensorimotor Network in Relapsing-Remitting Multiple Sclerosis: Evidence from a Centrality Analysis
Source: PLoS One. 2015 Jun 25;10(6):e0130524. doi: 10.1371/journal.pone.0130524 (PMC4482320; doi:10.1371/journal.pone.0130524)
Supplement: S5 Table — (DOC) [file pone.0130524.s012.doc]

**S5 Table. Altered centrality associated with the clinical metrics in the remitting patients.**

| Regions | Disease duration | | | EDSS | | | TWMLL | | | BPF | | |
| --- | --- | --- | --- | --- | --- | --- | --- | --- | --- | --- | --- | --- |
|  | *R^2^* | *P* | *β* | *R^2^* | *P* | *β* | *R^2^* | *P* | *β* | *R^2^* | *P* | *β* |
| Altered degree centrality in the remitting patients | | | | | | | | | | | | |
| Left fO | 0.010 | 0.576 | -0.101 | 0.003 | *0.759* | *0.055* | *0.049* | 0.209 | 0.211 | 0.000 | 0.958 | 0.009 |
| left OP/Ins | 0.003 | 0.782 | 0.505 | 0.002 | *0.799* | *-0.045* | *0.004* | 0.728 | 0.064 | **0.139** | **0.030** | **0.373** |
| Left MCC | 0.034 | 0.306 | 0.184 | 0.077 | *0.112* | *0.277* | *0.002* | 0.819 | -0.041 | 0.032 | 0.315 | 0.178 |
| Right SMA | 0.011 | 0.554 | 0.107 | 0.004 | *0.711* | *-0.066* | *0.000* | 0.989 | 0.002 | **0.139** | **0.030** | **0.373** |
| Left IPL | 0.011 | 0.553 | -0.107 | 0.0005 | *0.904* | *-0.022* | *0.016* | 0.476 | -0.126 | 0.001 | 0.889 | -0.025 |
| Right M1 | 0.021 | 0.419 | -0.146 | **0.162** | **0.018** | **0.403** | *0.052* | 0.192 | -0.229 | 0.016 | 0.471 | -0.128 |
| Left PMd | 0.101 | 0.071 | 0.318 | 0.079 | *0.107* | *0.281* | *0.005* | 0.705 | -0.067 | 0.001 | 0.849 | 0.034 |
| Left SPL | 0.043 | 0.248 | 0.207 | 0.004 | *0.720.* | *0.064* | *0.088* | 0.088 | 0.297 | 0.006 | 0.660 | -0.078 |
| Altered eigenvector centrality in the remitting patients | | | | | | | | | | | | |
| Left fO | 0.0004 | 0.916 | 0.019 | 0.0007 | *0.962* | *-0.009* | *0.008* | 0.625 | 0.087 | 0.000 | 0.974 | -0.006 |
| Left IPL | 0.039 | 0.268 | -0.198 | **0.198** | **0.008** | **0.445** | 0.036 | 0.279 | -0.191 | 0.010 | 0.571 | -0.101 |
| Bilateral MCC | 0.004 | 0.722 | 0.064 | 0.042 | 0.244 | 0.205 | 0.011 | 0.551 | 0.106 | 0.003 | 0.743 | -0.058 |
| Right SMA | 0.0009 | 0.958 | -0.010 | 0.015 | 0.483 | 0.124 | 0.001 | 0.853 | 0.033 | 0.009 | 0.602 | -0.093 |
| Left PostG | 0.000 | 0.997 | 0.001 | 0.004 | 0.714 | 0.065 | 0.005 | 0.699 | 0.059 | 0.216 | 0.220 | 0.216 |
| Right SPL | 0.046 | 0.229 | 0.215 | 0.038 | 0.272 | 0.194 | 0.040 | 0.257 | 0.200 | 0.038 | 0.271 | -0.194 |
| Right M1 | 0.002 | 0.785 | -0.049 | 0.006 | *0.661* | *0.078* | *0.005* | 0.680 | -0.073 | 0.009 | 0.589 | -0.096 |
